# Caseous Calcifications of Mitral Annulus as an Unusual Cause of Cardioembolic Stroke in a 40-Year-Old Man

**DOI:** 10.7759/cureus.3015

**Published:** 2018-07-20

**Authors:** Jian Liang Tan, Jonathan Finkel, Charles Geller

**Affiliations:** 1 Internal Medicine, Crozer-Chester Medical Center, Upland, USA; 2 Cardiology, Crozer-Chester Medical Center, Upland, USA; 3 Department of Cardiothoracic Surgery, Crozer Chester Medical Center, Upland, USA

**Keywords:** caseous calcifications of mitral annulus, cardioembolic stroke, transthoracic echocardiography, transesophageal echocardiography

## Abstract

Caseous calcification of mitral annulus (CCMA) is a rare variant of mitral annular calcification. Previously thought to have been a benign condition, CCMA may be a potential source of cardioembolic stroke. We present a case of a 40-year-old man with end-stage renal disease on hemodialysis and hypertension who presented with acute onset of visual blurring and headache and was diagnosed with cardioembolic stroke secondary to CCMA. It is imperative for the echocardiographers to recognize the typical features of CCMA and to differentiate it from other common causes for appropriate intervention.

## Introduction

Caseous calcifications of mitral annulus (CCMA) is a rare form of mitral annular calcification (MAC), with a prevalence of 0.6%-2.3%. It is typically diagnosed via an echocardiographic imaging [1]. Such echocardiographic lesion may masquerade as intracardiac tumors (myxoma, hemangioma, or leiomyosarcoma), thrombi, cysts, myocardial abscesses, or vegetations (inflammatory or infectious in origin) [1]. The aim of this report is to make clinicians aware of the typical echocardiographic appearance of CCMA to avoid diagnostic error and for appropriate intervention.

## Case presentation

A 40-year-old man with a history of end-stage renal disease on hemodialysis, hypertensive cardiomyopathy, and poorly controlled hypertension presented to the emergency department with a sudden onset of 48-hour right visual blurring and headache. On physical examination, he was alert, oriented to time, place and person, with a blood pressure of 200/124 mmHg, and a heart rate of 88 beats/minute. Neurologic examination was only significant for decreased right visual acuity. The electrocardiogram revealed normal sinus rhythm.

**Figure 1:**
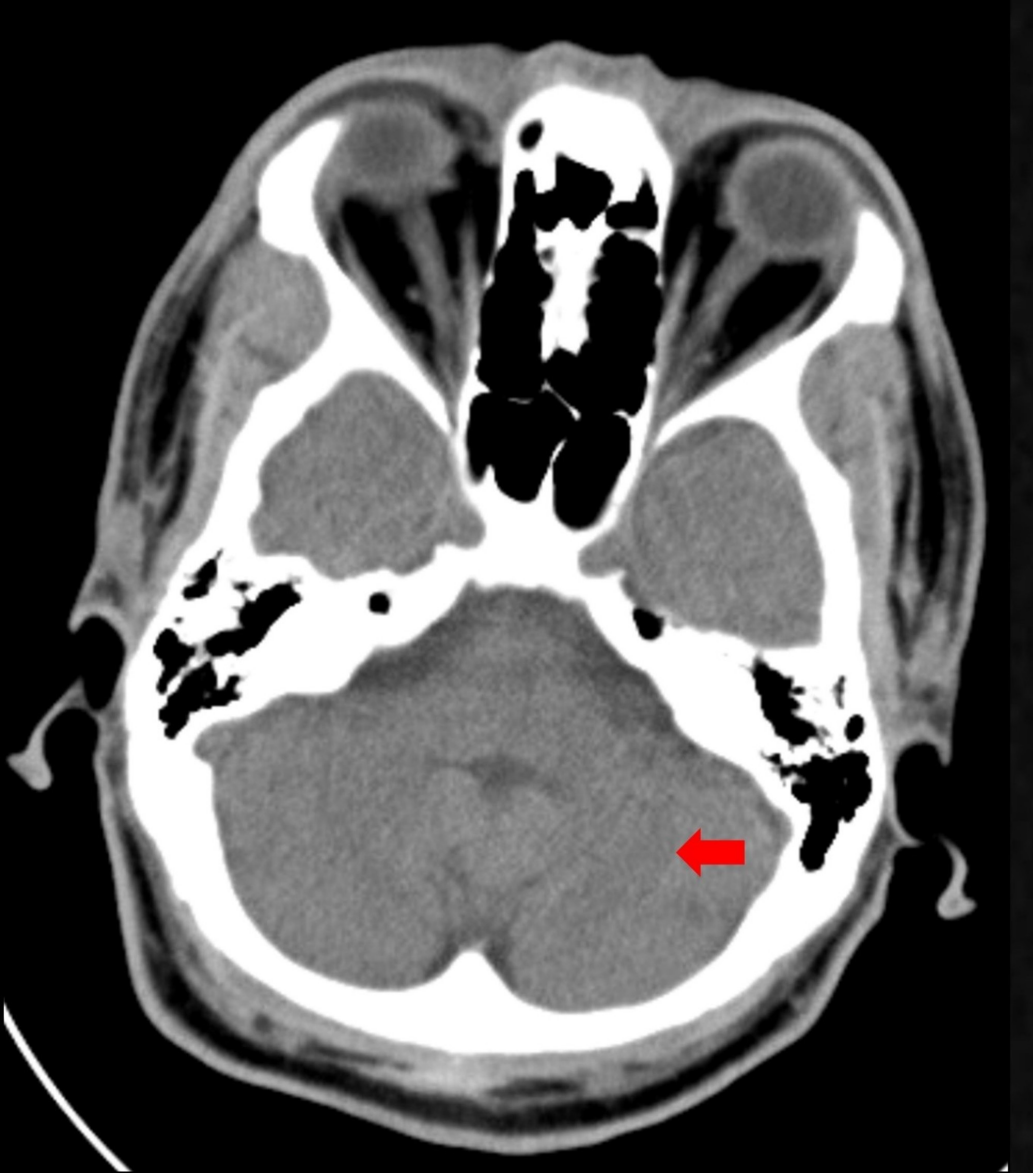
Noncontrast head computed tomography scan. Computed tomography of the head showed subtle focal area of hypoattenuation (red arrow) in the left cerebellar hemisphere.

Computed tomography of the head revealed focal area of hypoattenuation in the left cerebellar hemisphere (Figure 1). 

**Figure 2:**
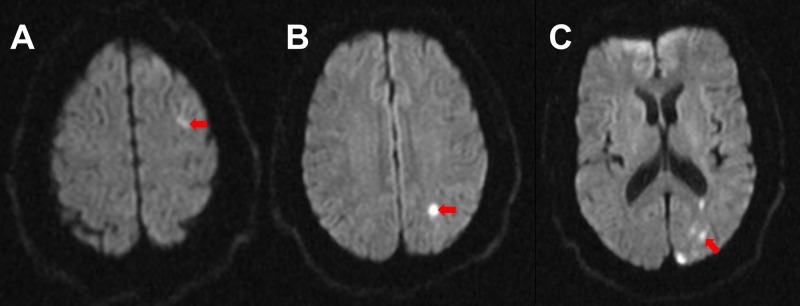
Brain magnetic resonance imaging. Diffusion-weighted magnetic resonance imaging of the brain revealed increase in signal intensity (red arrows) within left frontal (A), temporal (B), and occipital lobes (C).

Magnetic resonance imaging of the brain revealed multiple new regions of restricted diffusion within the left frontal, parietal and occipital lobes, consistent with an embolic stroke (Figure 2). 

**Figure 3:**
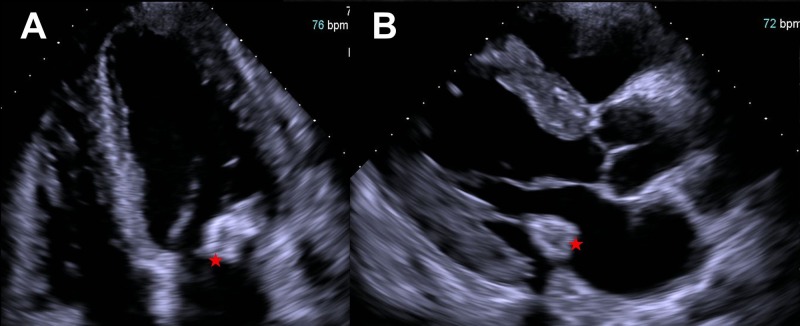
Two-dimensional transthoracic echocardiography. Two-dimensional transthoracic echocardiography - apical four chamber (A) view and long-axis parasternal (B) view demonstrated a nonmobile calcified mass (2.45 cm x 1.60 cm) located over the posterior mitral annulus, with central areas of echolucency (red asterisks) extending into the mitral inflow area.

A carotid duplex ultrasound was unremarkable for carotid artery stenosis. A two-dimensional transthoracic echocardiography revealed a large calcified mass measuring 24.5 mm x 16.0 mm (Figure 3, asterisks; Video 1).

**Video 1:** Two-dimensional transthoracic echocardiography.

**Figure 4:**
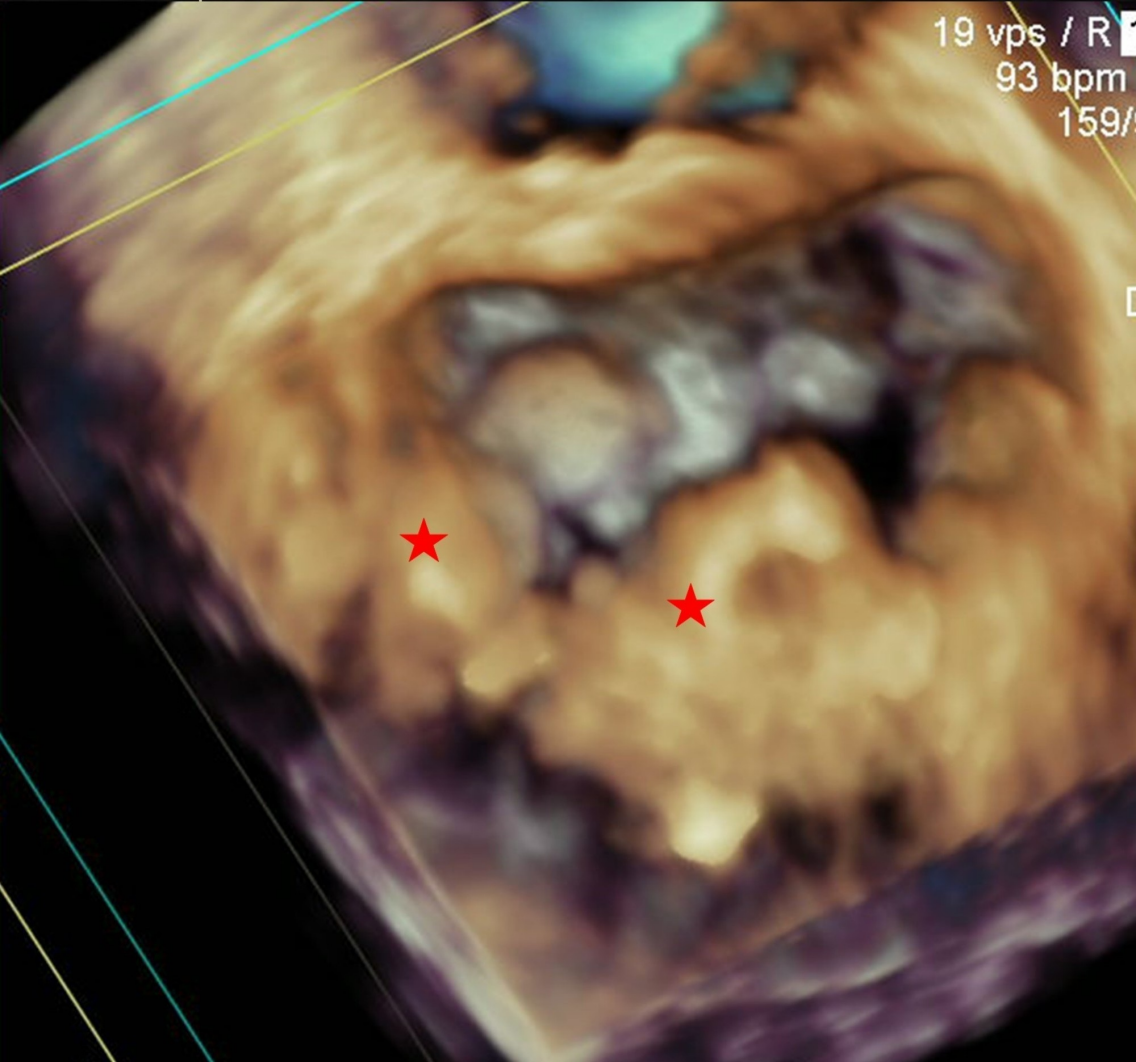
Three-dimensional transesophageal echocardiography. En face view of mitral valve showed two discrete nonmobile calcified masses (red asterisks), involving at least 50% of the posterior mitral valve annulus, with central areas of echolucency resembling liquefaction necrosis.

A three-dimensional transesophageal echocardiogram of the mitral valve revealed two discrete nonmobile calcified masses, with central areas of echolucency consistent with CCMA (Figure 4, asterisks; Video 2).

**Video 2:** Three-dimensional transesophageal echocardiography.

As no other potential sources of embolism were identified, CCMA lesion was postulated as the possible source of embolism. Dual antiplatelet therapy (aspirin 81 mg daily and clopidogrel 75 mg daily) and high-intensity atorvastatin 80 mg daily were initiated. Surgical excision of the CCMA lesion was considered but not performed immediately due to the potential risk of hemorrhagic conversion of the ischemic stroke. 

## Discussion

We described a case of a young adult presenting with right visual blurring and headache and was found to have CCMA as an unusual cause of cardioembolic stroke. Mitral annular calcification (MAC) is a chronic degenerative process occurring within the cardiovascular fibrous skeleton and mainly involves the posterior mitral annulus [1]. CCMA occurs as a sequela of the central liquefaction of MAC [1]. The prevalence is higher in elderly women, hypertensive patients, or chronic kidney disease patients with altered calcium-phosphate metabolism [2]. CCMA is typically diagnosed via an echocardiographic imaging, and appears as a round, large echo-dense mass with smooth borders, without acoustic shadowing, located in the mitral annular area, and with central areas of echolucencies due to liquefaction necrosis [1]. Previously thought to have been a benign condition, CCMA may be a potential source of cardioembolic stroke. Possible mechanisms for cerebral embolism in CCMA include embolization of small calcified particles, thrombus formation, or fistulization of caseous necrosis into the lumen of the left ventricle [3].

There is currently no consensus on the optimal management of CCMA. However, a conservative management strategy, which involved a follow-up echocardiography of the CCMA lesion is usually preferred. CCMA may be a dynamic process as it can resolve spontaneously or recur even after surgical excision [1, 4-5]. Surgical intervention may be considered for a patient with embolic phenomena, valvular dysfunction or to rule out the possibility of a tumor [5]. Our case had demonstrated CCMA as a potential source of the embolic stroke, and the surgical intervention was planned once the patient returned to the clinic for a follow-up with the cardiothoracic surgeon.

## Conclusions

The differential diagnosis of intracardiac lesion remains broad. It is prudent for echocardiographers, cardiologists, and cardiothoracic surgeons to maintain a high index of suspicion and be familiar with the echocardiographic features of CCMA to avoid diagnostic errors as clinical management varies widely.
